# Authentication and Quantitative Analysis of Adulteration in Ziziphi Spinosae Semen Based on Near-Infrared Spectroscopy and Chemometrics

**DOI:** 10.3390/foods15203690

**Published:** 2026-10-16

**Authors:** Yanyan Zhang, Xiang Pu, Zhiliang Fan, Yuexing Li, Yihui Chai

**Affiliations:** School of Basic Chinese Medicine (School of Qihuang Medicine), Guizhou University of Traditional Chinese Medicine, Guizhou 550025, China; yanyanzhang064@163.com (Y.Z.); puxiang0318@163.com (X.P.); zl_fan@163.com (Z.F.); 15236036020@163.com (Y.L.)

**Keywords:** ZSS, Fourier transform spectroscopy, near-infrared spectroscopy, chemometrics, DD-SIMCA, authenticity identification

## Abstract

Ziziphi Spinosae Semen (ZSS) is a traditional Chinese medicinal material used for both medicinal and dietary purposes, but substitution and adulteration can compromise its authenticity and quality. Ten batches of authentic ZSS and one source batch of each of five adulterant materials were used to prepare 325 laboratory samples. This study aimed to develop a rapid method for ZSS identification and adulteration quantification using Fourier transform near-infrared spectroscopy (FT-NIR). Spectral distributions of six sample classes were analyzed using PCA-X, PLS-DA, and OPLS-DA, and the classification performance of SVM, DA, ANN, and KNN was compared. DD-SIMCA was used for one-class authentication, while PLSR was applied to quantify five adulterants using 15 spectral processing approaches. In the internal test set, SVM achieved 98.0% accuracy and 98.4% balanced accuracy. Within the laboratory samples studied, DD-SIMCA achieved 100% accuracy, sensitivity, and specificity. Across the five selected PLSR models, prediction-set R^2^ ranged from 0.9272 to 0.9940 and RMSEP from 1.36 to 4.33 percentage points of adulterant mass fraction. These results demonstrate the feasibility of combining FT-NIR with chemometric methods for rapid ZSS classification, authenticity assessment, and adulteration-level prediction. Independent validation with additional source batches is required before practical application.

## 1. Introduction

Ziziphi Spinosae Semen (ZSS), the dried mature seed of *Ziziphus jujuba* Mill. var. *spinosa* (Bunge) Hu ex H.F. Chow of the family Rhamnaceae, is traditionally used in China as both medicine and food [1]. ZSS is mainly distributed in northern China, and its medicinal resources have long been obtained primarily through wild harvesting [2]. ZSS contains various constituents, including flavonoids, saponins, and organic acids, and exhibits pharmacological activities such as sedative–hypnotic, antidepressant, and anxiolytic effects; it is widely used in traditional Chinese medicine preparations and related health products [3,4].

In recent years, the price of ZSS has continued to rise because of factors such as declining wild resources and increasing market demand. During market circulation, low-cost seeds such as Ziziphi Mauritianae Semen (ZMS), Hovenia dulcis seed (HD), and Lens culinaris seeds (LCS) may be substituted for or mixed into ZSS. These counterfeit materials resemble ZSS in appearance but differ in botanical origin and chemical composition. Their substitution for, or admixture with, ZSS may affect the quality of the medicinal material and the intended performance of related products. Moreover, ZSS is often ground during processing and use, diminishing the shape, color, and texture features on which visual identification relies [5]. Current approaches to ZSS authentication include macroscopic and microscopic examination, chromatographic analysis, metabolomics, and molecular identification [3,5,6,7]. These methods provide evidence based on morphology, chemical composition, or genetic information. However, some require sample extraction, chromatographic separation, or DNA amplification, which can limit their suitability for rapid screening of large numbers of samples. Authentication and adulteration analysis of ZSS are therefore important for raw-material quality control.

FT-NIR can rapidly characterize overtone and combination-band absorptions of hydrogen-containing groups, such as C–H, O–H, and N–H, in samples and offers rapid analysis, simple operation, and no requirement for chemical reagents [8]. When combined with spectral preprocessing and chemometric methods, it can extract information related to sample class and constituent content from complex, overlapping spectral bands and has been widely used for the identification and quality evaluation of traditional Chinese medicines and powdered products [9]. A previous study reported a prediction-set identification rate of 83.72% when FT-NIR combined with SVM was used to classify the geographical origins of *Angelica sinensis*; when PLSR was used to predict ferulic acid and Z-ligustilide contents, the prediction-set R^2^ values were 0.9118 and 0.9206, respectively [10]. In a study of *Panax notoginseng* powder, a one-class model was used to screen adulterated samples and achieved an overall identification accuracy of 96.67%, demonstrating the feasibility of screening for non-target adulteration [11]. Quantitative models were also developed for saffron samples separately adulterated with lotus stamens and corn stigmas, and model prediction performance improved after informative spectral variables were selected [12]. These studies indicate that NIR spectroscopy combined with chemometrics can be used for medicinal-material classification, adulteration screening, and quantitative analysis. FT-NIR therefore provides a basis for developing a rapid method to assess the authenticity of powdered ZSS. However, multiclass models discriminate only among predefined classes. An adulterant absent from the training set may still be assigned to a known class, making it difficult to determine whether a test sample conforms to the overall spectral characteristics of authentic ZSS [13,14].

Data-driven soft independent modeling of class analogy (DD-SIMCA) establishes an acceptance region from the spectral distribution of target-class samples. It can assess whether a test sample conforms to the target class without requiring a separate class model for every potential adulterant [15,16]. To the best of our knowledge, this is the first application of DD-SIMCA to assess whether powdered ZSS conforms to the overall spectral characteristics of authentic ZSS, complementing the identification of predefined classes. The study included LCS, ZMS, and HD as commonly reported counterfeit materials. Adzuki bean (AB) and Scarlet runner bean (SRB) were included based on observations of their use as ZSS adulterants in the markets surveyed. These observations do not establish the prevalence of such adulteration in the broader market. After grinding and sieving, the samples were loaded directly for FT-NIR diffuse-reflectance measurement without solvent extraction or reagent treatment. Using the same spectral dataset, we developed models for multiclass classification, DD-SIMCA assessment of conformity to authentic ZSS, and PLSR prediction of adulterant mass fraction. We then evaluated the feasibility of this workflow for authenticity screening and adulteration quantification in the powdered samples examined.

## 2. Materials and Methods

### 2.1. Sample Sources

Ten batches of ZSS and one batch each of ZMS, HD, and LCS were purchased from the Bozhou medicinal material market, Bozhou, Anhui Province, China. One batch each of AB and SRB was purchased from a local market, Guiyang, Guizhou Province, China. The raw materials were identified by macroscopic examination of intact seeds before grinding. Professor Ning Ding of the Guizhou University of Traditional Chinese Medicine (Guiyang, Guizhou, China) examined the shape, size, color, and surface characteristics of each batch and found them consistent with the declared species. Each purchased batch had a corresponding identification number; details are provided in Supplementary Table S1.

### 2.2. Sample Preparation

To evaluate model performance across different adulteration levels, laboratory mixtures were prepared at adulterant mass fractions of 10–50%, in 10-percentage-point increments, based on the experimental design of a previous study [17]. These mixtures were used for model development and performance evaluation. Each raw material was ground separately using a YXA-5 grinder (VRera, Changzhou, China) and passed through a No. 2 sieve (24 mesh; nominal aperture, 0.850 mm; Lichen, Shaoxing, China). Material retained on the sieve was reground and sieved again, and the sieved powder was collected for subsequent experiments. No additional drying treatment was applied to the purchased materials. After grinding and sieving, the powders were stored in sealed bags under dry, light-protected conditions until spectral acquisition.

Separate laboratory mixtures were prepared by combining each of ZMS, HD, LCS, AB, and SRB with powder from the ten ZSS batches. Each mixture had a total mass of 5.00 g. The ZSS and adulterant powders were weighed separately using a dual-range electronic analytical balance (BP211d; Sartorius, Göttingen, Germany). At adulterant mass fractions of 10%, 20%, 30%, 40%, and 50%, the adulterant masses were 0.50, 1.00, 1.50, 2.00, and 2.50 g, respectively; the corresponding ZSS masses were 4.50, 4.00, 3.50, 3.00, and 2.50 g. For each adulterant, 10 samples were prepared at each adulteration level, yielding 250 samples with graded adulteration levels. In addition, 50 unadulterated ZSS samples were prepared, together with five control samples for each adulterant. A total of 325 samples were obtained. The raw material is shown in Figure 1A, and the blended sample is shown in Figure 1B. Detailed sample information is provided in Supplementary Table S2.

### 2.3. FT-NIR Spectral Acquisition

Spectra were acquired using an Antaris II Fourier transform near-infrared spectrometer (Thermo Fisher Scientific, Waltham, MA, USA) with the accompanying software for instrument control and data acquisition. After instrument calibration, samples were measured sequentially in sample-number order. For each sample, approximately 3 g of powder was weighed into the cylindrical glass sample tube supplied with the instrument. The tube was gently shaken, and the powder surface was leveled to promote uniform packing. Spectra were collected over 10,000–4000 cm^−1^ at a spectral resolution of 8 cm^−1^. A background spectrum was collected before each sample was loaded. Sixty-four consecutive scans were averaged to obtain one spectrum per sample. Each spectrum comprised 1557 spectral variables and was expressed as log(1/R), where R denotes relative reflectance. All measurements were conducted at an ambient temperature of 25 ± 2 °C and a relative humidity of 50% ± 5%.

### 2.4. FT-NIR Spectral Preprocessing

Near-infrared spectra are susceptible to non-target variation caused by baseline drift, random noise, and light scattering. This study compared raw spectra (RAW) with Savitzky–Golay smoothing (SG), standard normal variate (SNV), multiplicative scatter correction (MSC), first derivative (1st Der), second derivative (2nd Der), and their combinations, comprising 15 data processing approaches in total [18]. SG was used to reduce random noise while preserving local spectral profiles; SNV and MSC were used to correct scattering variation caused by differences in particle size and surface diffuse reflectance; and derivative processing was used to correct baselines and enhance the resolution of overlapping bands [19].

### 2.5. Multivariate Statistical Analysis

Unsupervised principal component analysis of the X matrix (PCA-X) and supervised partial least squares discriminant analysis (PLS-DA) and orthogonal partial least squares discriminant analysis (OPLS-DA) were used to examine the clustering and separation trends of the six sample classes. PCA-X was used to extract the main spectral variation without incorporating class information. In addition, separate PCA-X models were developed for each of the six sample classes to characterize within-class spectral variation. PLS-DA constructs a supervised classification model by maximizing the covariance between spectral variables and class variables. OPLS-DA separates predictive variation associated with class membership from systematic variation orthogonal to class membership, thereby improving model interpretability [20]. The number of components retained in the PCA-X, PLS-DA, and OPLS-DA models was determined using the Autofit function in SIMCA 14.1 (Umetrics, Umeå, Sweden). Model fit and predictive ability were evaluated using the coefficient of determination (R^2^) and the cumulative cross-validated predictive ability parameter (Q^2^), respectively, and two- and three-dimensional score plots were generated.

To assess the association between class labels and the spectra, separate permutation tests with 200 permutations each were performed for the PLS-DA and OPLS-DA models. For each permutation, class labels were randomly shuffled, and the model was refitted; R^2^Y and Q^2^ values for the permuted model were recorded and compared with those of the original model. When the Q^2^ values of the permuted models were generally lower than those of the original model, and the Q^2^ regression line had a negative intercept, these results were interpreted as supporting evidence of a lower risk of overfitting.

### 2.6. Machine Learning Classification Models

Support vector machine (SVM), discriminant analysis (DA), artificial neural network (ANN), and K-nearest neighbor (KNN) were used to compare the recognition performance for the six sample classes [21]. The 325 samples were divided at a 7:3 ratio into a training set of 227 samples and an internal test set of 98 samples. The 1557 spectral variables were used as input features, and the six sample-class labels were used as the output variable. The classes were defined according to sample authenticity and adulterant type. The ZSS class comprised 50 unadulterated ZSS samples. Each of the other five classes comprised 50 ZSS mixtures containing 10–50% of a given adulterant and five corresponding pure-adulterant controls, for a total of 55 samples per class. These five adulterant-system classes were labeled LCS, AB, SRB, ZMS, and HD, respectively. The training set was used for model fitting and ten-fold cross-validation. The internal test set was not used for model training or parameter optimization and was reserved for evaluating the classification performance of the final models [22,23]. Confusion matrices were constructed from the true and predicted class labels to show correct classifications and misclassifications for each class. Overall accuracy, class-wise sensitivity (recall), precision, and F1 score were calculated from the internal test-set confusion matrices.

### 2.7. DD-SIMCA One-Class Classification Model

DD-SIMCA was used to establish an authenticity model with authentic ZSS as the target class [24]. The 50 authentic ZSS samples were divided using the Kennard–Stone algorithm at a 6:2:2 ratio into a training set (*n* = 30), a validation set (*n* = 10), and an internal test set (*n* = 10) [16]. Only authentic samples in the training set were used to develop the DD-SIMCA model. The model used autoscaling and classical parameter estimation, with both the type I error rate and the outlier significance level set to 0.01. Distribution parameters for the score distance and orthogonal distance were estimated from the training set. The two distances were then standardized and combined into a total distance, and the target-class acceptance boundary was determined using a chi-square critical value. The validation set was used to select model parameters, primarily by requiring acceptance of all authentic validation samples while also considering rejection of non-target samples. Four principal components were ultimately retained. The internal test set was not used for model development or parameter selection and was reserved for the final performance evaluation. Samples within the acceptance region were classified as the target class, whereas those outside the acceptance region were classified as non-target samples.

### 2.8. Development and Evaluation of the PLSR Models

Partial least squares regression (PLSR) was used to model the relationship between the near-infrared spectra and adulterant mass fraction. Separate PLSR datasets were constructed for the ZSS–LCS, ZSS–AB, ZSS–SRB, ZSS–ZMS, and ZSS–HD systems. Each dataset comprised 50 unadulterated ZSS samples, 50 mixtures containing 10–50% adulterant by mass, and five pure-adulterant controls, for a total of 105 samples. Reference values of 0% and 100% were assigned to the unadulterated ZSS samples and pure-adulterant controls, respectively. The sample allocation for each system is detailed in Supplementary Table S4.

Before spectral preprocessing and PLSR modeling, each 105-sample dataset was divided using the Kennard–Stone algorithm at a 7:3 ratio into a calibration set (*n* = 74) and a prediction set (*n* = 31). Under each preprocessing condition, 10-fold cross-validation of the calibration set was used to assess model performance across different numbers of latent variables (LVs), and the number of LVs corresponding to the minimum root mean square error of cross-validation (RMSECV) was selected as optimal. The models were comprehensively evaluated using the coefficient of determination for calibration (R^2^c), root mean square error of calibration (RMSEC), mean absolute error of the calibration set (MAEc), ratio of performance to deviation for the calibration set (RPDc), coefficient of determination for prediction (R^2^p), root mean square error of prediction (RMSEP), mean absolute error of the prediction set (MAEp), and ratio of performance to deviation for the prediction set (RPDp). The final model for each system was selected primarily on the basis of predictive performance and model complexity. When predictive performance was similar, the model with fewer LVs was preferred. RMSEC, RMSEP, MAEc, and MAEp were expressed in percentage points of adulterant mass fraction.

### 2.9. Data Analysis

Spectral preprocessing and the PCA, PLS-DA, and OPLS-DA analyses were conducted primarily in SIMCA 14.1 (Umetrics, Umeå, Sweden). Machine learning classification, DD-SIMCA, and PLSR were performed in MATLAB R2024b (MathWorks, Natick, MA, USA). Figures were generated using SIMCA 14.1 and MATLAB R2024b.

## 3. Results

### 3.1. Near-Infrared Spectral Characteristics

The experimental workflow is shown in Figure 1C. The raw FT-NIR spectra of the 325 samples are shown in Figure 2A. The six sample classes showed similar overall profiles over the range of 10,000–4000 cm^−1^ but differed in baseline level, absorption intensity, and local spectral shape, with the main differences occurring at 8300–8200, 7100–6800, 5900–5600, 5300–5100, and 4800–4300 cm^−1^. The mean spectrum of each class (Figure 2B) showed distinct absorption peaks at approximately 8285, 6800, 5809, 5164, and 4597 cm^−1^. The absorption band near 6800 cm^−1^ was relatively broad.

The spectra after MSC preprocessing are shown in Figure 2C,D. Compared with the raw spectra, the preprocessed spectral curves were more tightly clustered, and scattering-induced baseline shifts and overall scale differences were substantially reduced, whereas the positions of the main absorption bands and the overall profiles remained essentially unchanged, indicating that MSC improved spectral comparability among samples. However, the mean spectra of the six sample classes still overlapped substantially after preprocessing, with only subtle differences in absorption intensity and local spectral shape at approximately 7100, 5900, 5300–5000, and 4800–4200 cm^−1^. Therefore, direct comparison of the spectral curves alone was insufficient to reliably distinguish the six sample classes, and chemometric methods were required to further extract class-related differences.

### 3.2. PCA-X, PLS-DA, and OPLS-DA Analyses

PCA-X, PLS-DA, and OPLS-DA models were established to examine the overall distributions and class separation trends of the six sample classes. The PCA-X model used five principal components and yielded R^2^X(cum) = 0.996. The six sample classes overlapped extensively in both the two- and three-dimensional score plots, with only a few samples showing dispersed distributions, and no clear class separation was observed (Figure 3A,D). Separate PCA-X analyses indicated that a small number of principal components captured the main spectral variation within each class. Detailed model parameters are provided in Supplementary Table S3.

Compared with PCA-X, the PLS-DA score plots showed some degree of class clustering and separation, whereas class separation was clearer with OPLS-DA (Figure 3B,C,E,F). Autofit retained 10 components for the PLS-DA model, yielding R^2^X(cum), R^2^Y(cum), and Q^2^(cum) values of 1.000, 0.688, and 0.666, respectively. For the OPLS-DA model, Autofit retained five predictive components and seven orthogonal components (5 + 7 + 0), with corresponding R^2^X(cum), R^2^Y(cum), and Q^2^(cum) values of 1.000, 0.722, and 0.703, respectively. The slightly higher R^2^Y(cum) and Q^2^(cum) values of OPLS-DA relative to PLS-DA indicated improved predictive ability, although some classes still overlapped.

After 200 permutation tests, the regression-line intercepts were R^2^ = 0.00604 and Q^2^ = −0.118 for the PLS-DA model and R^2^ = 0.00833 and Q^2^ = −0.094 for the OPLS-DA model (Figure 3G,H). The permuted models yielded lower R^2^ and Q^2^ values than the corresponding original models, and both Q^2^ intercepts were negative. These findings provided supporting evidence of a lower risk of overfitting in the two supervised models.

### 3.3. Machine Learning Classification

SVM classifies high-dimensional spectral data by identifying an optimal decision boundary [25]. DA assigns classes according to the statistical distributions of spectral variables within each class [26]. ANN captures complex nonlinear relationships between spectra and sample classes [27]. KNN classifies samples based on local spectral similarity and can reflect class clustering in the local spectral space [28]. The classification results are shown in Figure 4A–H. The training-set accuracies of SVM, DA, ANN, and KNN were 100.0%, 96.9%, 96.9%, and 79.3%, respectively; their internal test-set accuracies were 98.0%, 95.9%, 96.9%, and 88.8%, respectively. SVM correctly classified 96 of the 98 internal test samples, compared with 94 of 98 for DA. SVM misclassified one LCS sample as SRB and one ZMS sample as ZSS; DA misclassified four samples, ANN three, and KNN 11 across multiple classes. The overall accuracies of all four models and the class-wise performance metrics for SVM and DA are provided in Supplementary Table S5. Both SVM and DA performed well on the internal test set, with numerically higher metrics for SVM.

### 3.4. DD-SIMCA Authenticity Discrimination

The DD-SIMCA model was established using 30 authentic ZSS samples, and four principal components were ultimately selected. In the acceptance plot, the horizontal axis, log(1 + h/h_0_), represents the score distance of a sample in the model space; the vertical axis, log(1 + v/v_0_), represents the orthogonal distance not explained by the model; and the green curve denotes the acceptance boundary. As shown in Figure 4I,J, authentic samples in the test and validation sets were mainly distributed at approximately 0–0.9 on the horizontal axis and 0.5–1.5 on the vertical axis, whereas the non-target samples were distributed at approximately 2.0–7.1 on the vertical axis. The score distances of the two sample groups overlapped to some extent, but the groups were clearly separated along the orthogonal-distance axis. In the internal test set, the model accepted all 10 authentic ZSS samples and rejected all 138 non-target samples, yielding sensitivity, specificity, and accuracy of 100.0%. In the validation set, it accepted all 10 authentic samples and rejected all 137 non-target samples; sensitivity, specificity, and accuracy were likewise 100.0%.

### 3.5. PLSR Quantitative Analysis

After the qualitative identification of ZSS and the five adulterants, separate PLSR models were established for the ZSS–LCS, ZSS–AB, ZSS–SRB, ZSS–ZMS, and ZSS–HD systems to predict the adulteration levels of the different adulterants. The RAW models yielded R^2^p values of 0.9080–0.9871 and RMSEP values of 1.75–4.87 percentage points of adulterant mass fraction, indicating that the raw spectra contained quantitative information related to adulteration level, although predictive performance varied among the systems. The modeling results obtained using the different preprocessing methods are shown in Table 1. Model selection was based primarily on the RMSEP of the prediction set, together with a comprehensive evaluation of R^2^p, MAEp, RPDp, and LV.

The effects of the preprocessing methods on model performance varied, and some methods did not markedly improve the prediction results. After screening, all five selected models used MSC alone or in combination with other preprocessing methods. Compared with the corresponding RAW models, the selected models showed higher R^2^p and RPDp values and lower RMSEP and MAEp values. MSC+1st Der was selected for ZSS–LCS (R^2^p = 0.9940), MSC+2nd Der for ZSS–AB (R^2^p = 0.9912), MSC+2nd Der for ZSS–SRB (R^2^p = 0.9272), MSC+2nd Der+SG for ZSS–ZMS (R^2^p = 0.9824), and MSC+1st Der+SG for ZSS–HD (R^2^p = 0.9888). Overall, the predicted and measured values of the five selected models were in good agreement (Figure 5A–E).

## 4. Discussion

ZSS has both medicinal and dietary value and is an important raw material for traditional Chinese medicine preparations and related products. Macroscopic identification relies on the external features of the seeds, whereas chromatographic and molecular methods provide evidence at different levels but generally require extraction, separation, or amplification. In a computer-vision study, color and texture features extracted from seed images enabled non-destructive classification of ZSS, ZMS, and Hovenia acerba seeds (HAS), with reported accuracies of 96–99% [29]. Another study combined transfer learning with multimodal fusion of hyperspectral image features and spectral information to identify ZSS samples adulterated with ZMS or HAS; the fusion model achieved an accuracy above 98% [18]. These findings demonstrate the discriminatory ability of image-based methods for the samples examined but do not establish equivalent performance for powdered samples.

FT-NIR can rapidly acquire spectral information from powdered samples and offers simple operation without solvent extraction [8]. Compared with image-based methods that rely on seed color and texture, FT-NIR can also be applied to powders in which these external features have become less distinct. Accordingly, this study examined powdered samples containing five adulterant materials and used the same spectral dataset for multiclass recognition, assessment of conformity to authentic ZSS, and prediction of adulterant mass fraction. The six classes overlapped extensively in the PCA-X score plots, indicating that the dominant spectral variation did not produce clear class separation. After class information was introduced, PLS-DA and OPLS-DA showed clearer separation trends. In the internal test set, SVM and DA correctly classified 96/98 and 94/98 samples, corresponding to accuracies of 98.0% and 95.9%, respectively. SVM had numerically higher balanced accuracy and macro-averaged F1 score than DA. DD-SIMCA accepted 10/10 authentic samples and rejected 138/138 non-target samples in the internal test set; in the validation set, it accepted 10/10 authentic samples and rejected 137/137 non-target samples. These findings show that the model rejected the non-target samples included in this study, but its performance against other adulterants remains to be evaluated. For the five selected PLSR models, prediction-set R^2^p ranged from 0.9272 to 0.9940, and RMSEP ranged from 1.36 to 4.33 percentage points of adulterant mass fraction, suggesting the feasibility of estimating adulteration within the sample set examined.

Near-infrared bands are broad and often overlap. The spectral differences observed near 8285, 6800, 5809, 5164, and 4597 cm^−1^ may therefore reflect combined variation in several constituents. The region near 8285 cm^−1^ lies within the second-overtone region of C–H stretching and may include contributions from lipids and other constituents [30]. The band near 5809 cm^−1^ is associated with the first overtone of C–H stretching and may reflect contributions from both lipids and carbohydrates [8]. The region near 6800 cm^−1^ lies within the first-overtone region of O–H stretching and may be influenced by water and other hydroxyl-containing constituents [31]. The band near 5164 cm^−1^ is consistent with a combination of O–H stretching and bending vibrations of water [32]. The region near 4597 cm^−1^ contains overlapping combination bands and may include information related to proteins and carbohydrates [33]. Given this overlap, these assignments indicate possible vibrational origins; no individual band can be treated as specific evidence for a particular constituent or for ZSS identity.

Absorption near 6800 and 5164 cm^−1^ suggests that moisture may have contributed to the spectral differences observed in this study [34]. Although the samples were stored under dry conditions, their moisture contents may still have differed. Because each adulterant was represented by only one batch, moisture-related differences could not be separated from differences among the materials. A contribution of moisture to the classification and quantitative results therefore cannot be excluded. After MSC preprocessing, differences in spectral baseline and overall intensity were reduced, and all five selected PLSR models used MSC alone or in combination with other methods. This is consistent with the role of MSC in reducing scattering differences among powders [35]. However, preprocessing effects varied among the adulteration systems, and the present results do not establish that MSC would remain the best choice for new source batches or data acquired using other instruments. Of the five systems, ZSS–SRB showed comparatively weaker prediction performance, with a prediction-set R^2^p of 0.9272 and an RMSEP of 4.33 percentage points, compared with RMSEP values of 1.36–2.48 percentage points for the other four systems. Differences in material composition or powder properties may have contributed to the larger error for ZSS–SRB, but the specific cause remains undetermined.

The 325 samples prepared in this study originated from ten ZSS batches and one batch of each of the five adulterant materials. The same adulterant batch was used at multiple mixture levels. Because the training, validation, and test sets were not partitioned by source batch, samples derived from the same batch may have occurred in different sets. The reported performance therefore mainly reflects these source materials under the laboratory preparation conditions used here. Differences in geographical origin, harvest season, moisture content, and particle size after grinding may introduce additional spectral variation. Moisture content and actual particle size were not measured separately. Although all samples were ground, sieved, stored, and measured using the same procedures, model stability after reloading samples or changing instruments has not been evaluated. Mixtures were prepared at adulterant mass fractions of 10–50%, with separate 0% authentic-ZSS and 100% pure-adulterant controls. Because no mixtures containing less than 10% adulterant were prepared, the present data do not establish detection performance below that level. A previous study developed global UV spectroscopic models for monitoring herbal medicine manufacturing by incorporating variation among process intermediates and systematically validating model performance, providing a methodological reference for extending spectroscopic models to production applications [36]. Future work should include materials from more sources and batches, lower-level mixtures, batch-grouped validation, market samples, and data acquired using different instruments to assess model applicability to new samples and measurement conditions.

## 5. Conclusions

This study developed a workflow combining FT-NIR spectroscopy, multivariate statistical analysis, multiclass machine learning, DD-SIMCA, and PLSR for ZSS authentication and prediction of adulterant mass fraction. Using the same spectral dataset, the models provided information on predefined-class recognition, conformity to authentic ZSS, and adulteration-level prediction, demonstrating the workflow’s potential within the laboratory-prepared samples examined. The results have not yet been validated using partitions grouped by source batch or evaluated with independent market or production-site samples. Future work should include authentic and adulterant materials from more sources and batches and assess model performance under actual market and production conditions before its suitability for raw-material acceptance and production-process monitoring can be established.

## Figures and Tables

**Figure 1:**
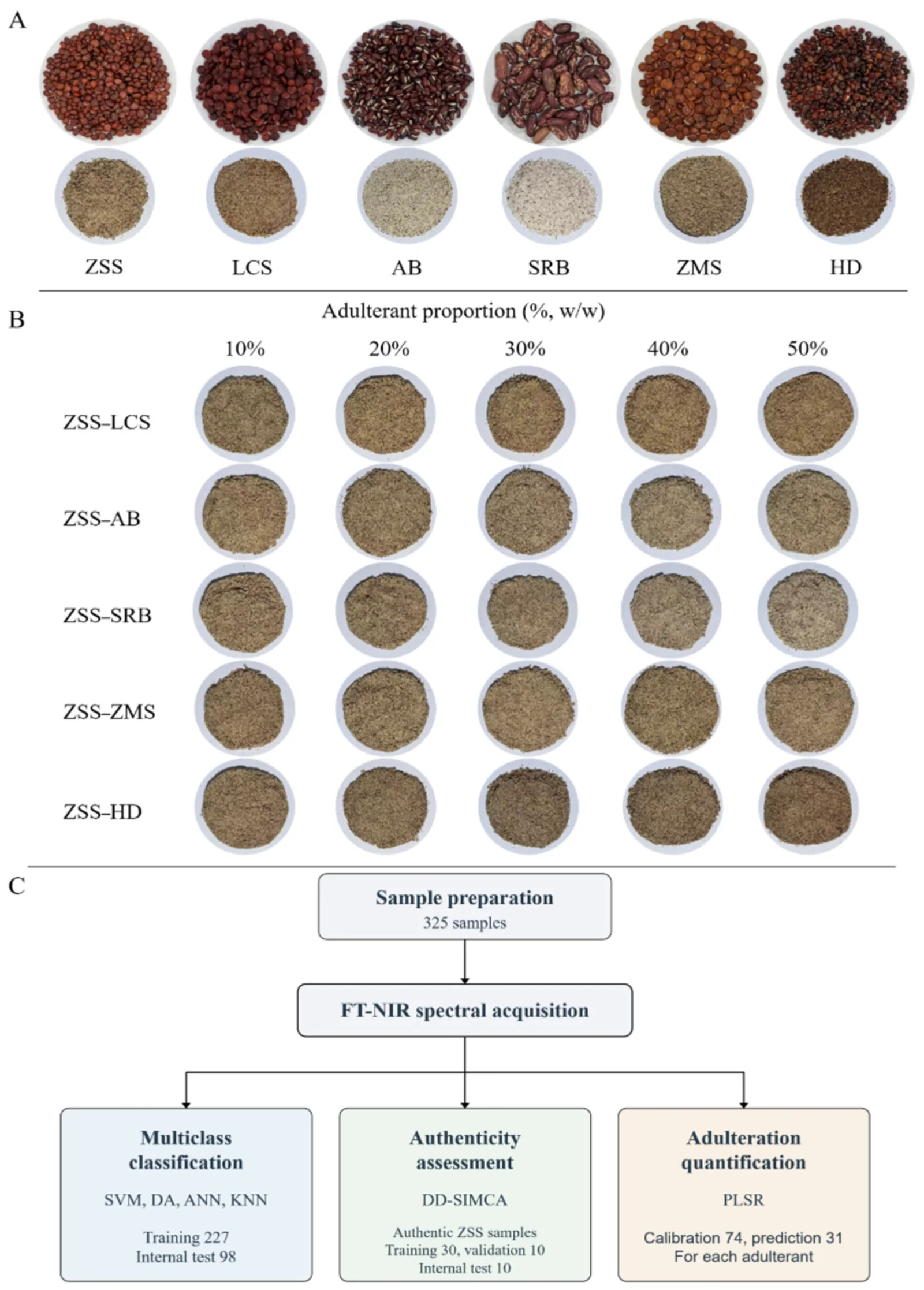
Sample appearance and experimental workflow: (**A**) original samples of ZSS and the five adulterants; (**B**) mixed samples prepared with the five adulterants at adulteration levels of 10–50%; (**C**) experimental workflow showing sample preparation.

**Figure 2:**
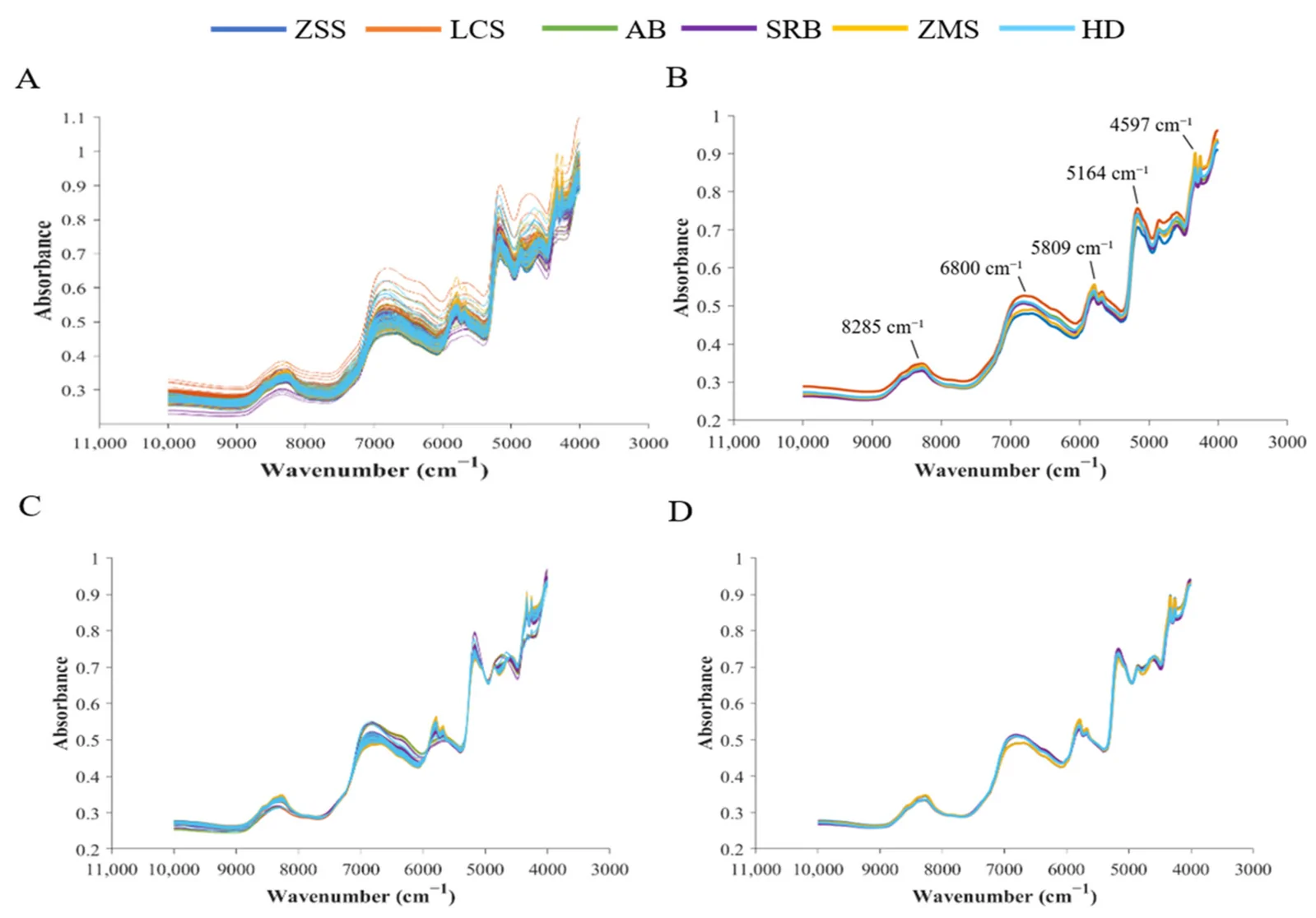
FT-NIR spectra of ZSS and the five adulterant classes: (**A**) raw spectra of all samples; (**B**) mean raw spectrum of each class; (**C**) spectra of all samples after MSC preprocessing; (**D**) mean spectrum of each class after MSC preprocessing.

**Figure 3:**
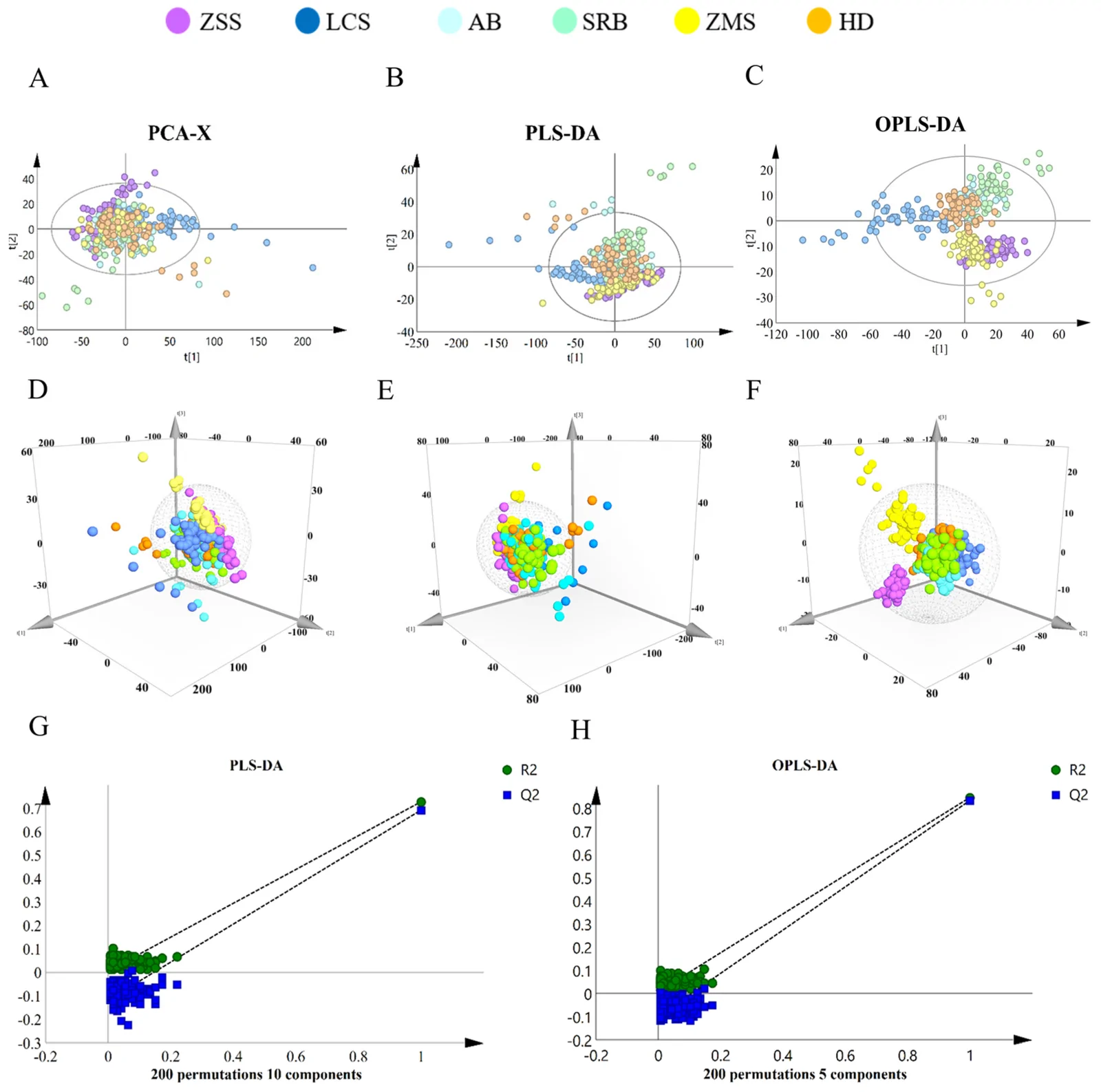
Multivariate statistical analysis of the six sample classes: (**A**,**D**) two- and three-dimensional PCA-X score plots; (**B**,**E**) two- and three-dimensional PLS-DA score plots; (**C**,**F**) two- and three-dimensional OPLS-DA score plots; (**G**,**H**) results of 200 permutation tests for the PLS-DA and OPLS-DA models, respectively. In both panels, the horizontal axis represents Corr(Y, Yperm), and the vertical axis shows R^2^Y(cum) and Q^2^(cum). The points at Corr(Y, Yperm) = 1 represent the original models; the remaining points represent the permuted models, and the lines are the corresponding regression lines.

**Figure 4:**
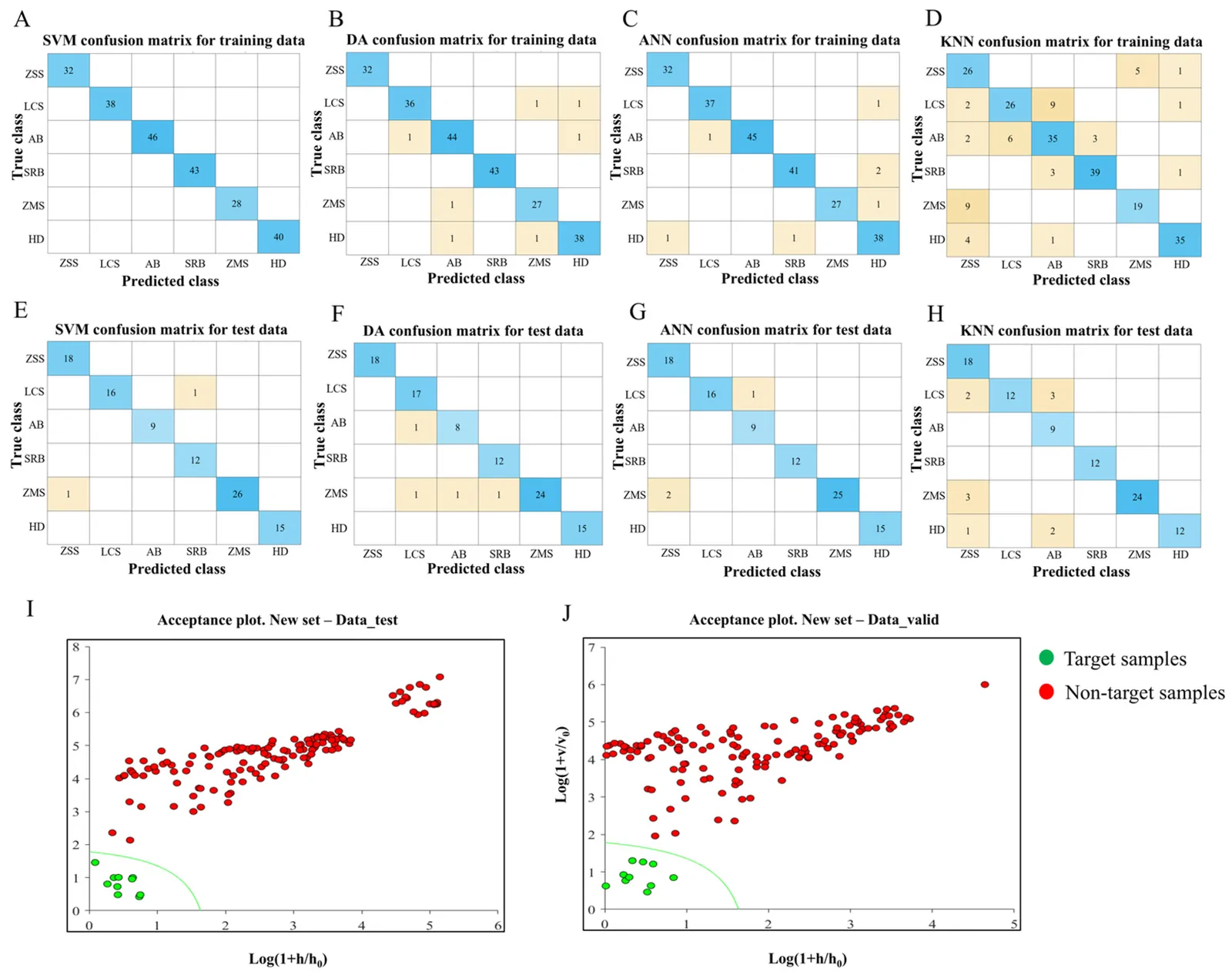
Machine learning classification results and DD-SIMCA acceptance plots: (**A**–**D**) training-set confusion matrices for the SVM, DA, ANN, and KNN models; (**E**–**H**) test-set confusion matrices for the SVM, DA, ANN, and KNN models; (**I**,**J**) DD-SIMCA acceptance plots for the test and validation sets. In the confusion matrices, rows represent the true classes and columns represent the predicted classes; blue diagonal cells indicate correct classifications, pale-yellow off-diagonal cells indicate misclassifications, and the numbers indicate the corresponding sample counts. In the DD-SIMCA acceptance plots, green points represent authentic ZSS samples, red points represent the five classes of non-target samples, and the green curve represents the boundary of the target-class acceptance region.

**Figure 5:**
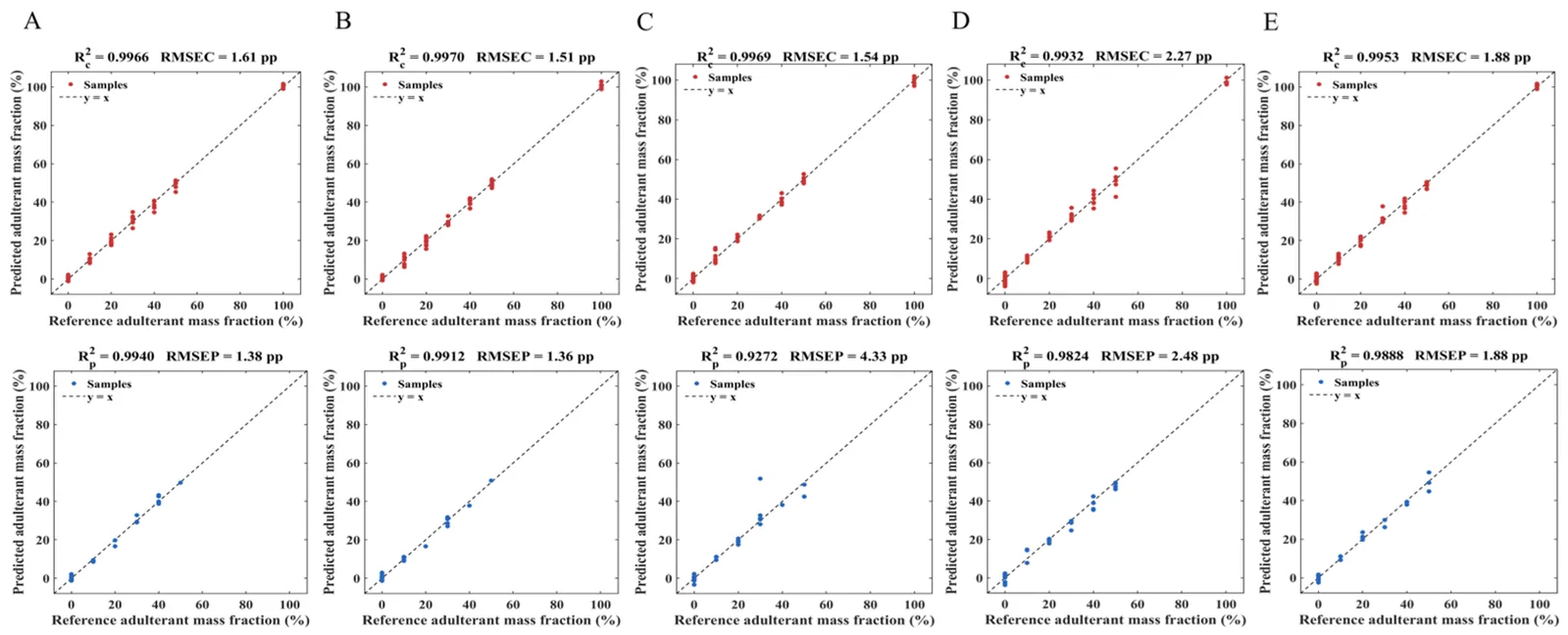
Relationships between reference and predicted values for the five selected PLSR models: (**A**) ZSS–LCS: MSC+1st Der; (**B**) ZSS–AB: MSC+2nd Der; (**C**) ZSS–SRB: MSC+2nd Der; (**D**) ZSS–ZMS: MSC+2nd Der+SG; (**E**) ZSS–HD: MSC+1st Der+SG. The upper row shows the calibration set, and the lower row shows the prediction set.

**Table 1:** Prediction results of the PLSR models for ZSS adulteration levels using different spectral preprocessing methods.

Methods		Calibration Set					Prediction Set			
		LV	RMSEC	R^2^c	MAEc	RPDc	RMSEP	R^2^p	MAEp	RPDp
ZSS–LCS	RAW	5	2.52	0.99	1.96	10.97	2.02	0.99	1.62	8.80
	1st Der+SG	3	2.26	0.99	1.76	12.22	1.66	0.99	1.22	10.90
	1st Der	4	1.92	1.00	1.47	14.36	1.53	0.99	1.16	11.64
	2nd Der+SG	3	1.75	1.00	1.32	15.83	1.81	0.99	1.37	10.08
	2nd Der	3	1.60	1.00	1.20	17.31	1.71	0.99	1.31	10.65
	MSC+1st Der+SG	4	1.59	1.00	1.21	17.39	1.45	0.99	1.06	12.31
	MSC+1st Der	3	1.61	1.00	1.16	17.13	1.38	0.99	1.03	12.89
	MSC+2nd Der+SG	2	1.60	1.00	1.10	17.30	1.53	0.99	1.13	11.65
	MSC+2nd Der	2	1.58	1.00	1.09	17.49	1.51	0.99	1.17	11.76
	MSC	5	1.81	1.00	1.39	15.24	1.68	0.99	1.31	10.64
	SNV+1st Der+SG	2	1.83	1.00	1.41	15.06	1.47	0.99	1.08	12.09
	SNV+1st Der	2	1.84	1.00	1.41	15.05	1.47	0.99	1.09	12.09
	SNV+2nd Der+SG	2	1.61	1.00	1.10	17.19	1.55	0.99	1.15	11.47
	SNV+2nd Der	2	1.59	1.00	1.10	17.38	1.54	0.99	1.20	11.57
	SNV	5	1.88	1.00	1.49	14.69	1.75	0.99	1.35	10.18
ZSS–AB	RAW	6	2.23	0.99	1.71	12.42	1.75	0.99	1.39	8.44
	1st Der+SG	3	2.43	0.99	2.00	11.40	1.72	0.99	1.41	8.52
	1st Der	4	2.17	0.99	1.75	12.73	1.82	0.98	1.42	8.01
	2nd Der+SG	2	2.34	0.99	1.78	11.80	1.54	0.99	1.14	9.42
	2nd Der	2	2.03	0.99	1.51	13.63	1.44	0.99	1.17	10.15
	MSC+1st Der+SG	3	1.62	1.00	1.30	17.06	1.32	0.99	1.12	11.01
	MSC+1st Der	3	1.30	1.00	1.05	21.25	1.41	0.99	1.17	10.37
	MSC+2nd Der+SG	2	1.54	1.00	1.17	17.91	1.41	0.99	1.07	10.29
	MSC+2nd Der	2	1.51	1.00	1.13	18.32	1.36	0.99	1.02	10.67
	MSC	8	1.45	1.00	1.10	19.06	1.39	0.99	1.13	10.44
	SNV+1st Der+SG	4	1.37	1.00	1.09	20.17	1.39	0.99	1.15	10.52
	SNV+1st Der	3	1.62	1.00	1.30	17.06	1.36	0.99	1.15	10.68
	SNV+2nd Der+SG	2	1.57	1.00	1.19	17.60	1.43	0.99	1.07	10.12
	SNV+2nd Der	4	0.75	1.00	0.57	36.97	1.69	0.99	1.37	8.74
	SNV	9	1.37	1.00	1.04	20.12	1.20	0.99	0.94	12.12
ZSS–SRB	RAW	4	2.64	0.99	2.13	10.52	4.87	0.91	3.22	3.31
	1st Der+SG	4	1.60	1.00	1.18	17.37	4.35	0.93	2.04	3.70
	1st Der	2	2.58	0.99	2.14	10.75	4.89	0.91	3.16	3.31
	2nd Der+SG	3	1.58	1.00	1.24	17.55	4.31	0.93	2.05	3.72
	2nd Der	2	2.23	0.99	1.85	12.43	4.70	0.91	3.06	3.43
	MSC+1st Der+SG	4	1.49	1.00	1.13	18.65	4.52	0.92	2.36	3.57
	MSC+1st Der	2	2.01	0.99	1.57	13.80	4.52	0.92	2.04	3.55
	MSC+2nd Der+SG	3	1.64	1.00	1.24	16.96	4.34	0.93	1.86	3.71
	MSC+2nd Der	3	1.54	1.00	1.16	18.01	4.33	0.93	1.87	3.71
	MSC	3	2.49	0.99	1.99	11.17	4.71	0.91	2.67	3.41
	SNV+1st Der+SG	2	2.09	0.99	1.64	13.26	4.53	0.92	2.10	3.55
	SNV+1st Der	2	2.05	0.99	1.61	13.53	4.52	0.92	2.08	3.56
	SNV+2nd Der+SG	3	1.64	1.00	1.25	16.89	4.35	0.93	1.87	3.70
	SNV+2nd Der	3	1.55	1.00	1.17	17.97	4.35	0.93	1.88	3.70
	SNV	3	2.40	0.99	1.91	11.56	4.86	0.91	2.78	3.31
ZSS–ZMS	RAW	8	2.53	0.99	2.03	10.87	3.04	0.97	2.53	6.79
	1st Der+SG	5	2.16	0.99	1.69	12.75	3.29	0.97	2.67	6.11
	1st Der	4	2.55	0.99	1.93	10.80	3.45	0.97	2.71	6.00
	2nd Der+SG	4	1.91	1.00	1.46	14.41	3.32	0.97	2.59	6.08
	2nd Der	3	2.61	0.99	2.08	10.53	3.88	0.96	3.22	5.21
	MSC+1st Der+SG	5	2.20	0.99	1.69	12.52	2.48	0.98	2.05	7.95
	MSC+1st Der	4	2.28	0.99	1.75	12.06	2.65	0.98	2.17	7.64
	MSC+2nd Der+SG	3	2.27	0.99	1.73	12.14	2.48	0.98	1.99	7.68
	MSC+2nd Der	3	2.12	0.99	1.59	12.96	2.80	0.98	2.34	6.94
	MSC	7	2.62	0.99	2.03	10.50	2.99	0.97	2.27	6.82
	SNV+1st Der+SG	4	2.39	0.99	1.84	11.53	2.75	0.98	2.23	7.35
	SNV+1st Der	4	2.56	0.99	1.91	10.74	3.11	0.97	2.52	6.21
	SNV+2nd Der+SG	3	2.27	0.99	1.73	12.14	2.49	0.98	1.99	7.67
	SNV+2nd Der	3	2.12	0.99	1.59	12.96	2.80	0.98	2.34	6.94
	SNV	7	2.62	0.99	2.03	10.51	3.02	0.97	2.29	6.76
ZSS–HD	RAW	7	2.66	0.99	1.97	10.36	2.53	0.98	1.87	7.06
	1st Der+SG	4	2.22	0.99	1.71	12.40	2.24	0.98	1.76	7.94
	1st Der	5	1.81	1.00	1.39	15.21	2.12	0.99	1.52	8.43
	2nd Der+SG	3	2.25	0.99	1.78	12.24	2.87	0.97	2.28	6.21
	2nd Der	3	1.90	1.00	1.49	14.52	3.04	0.97	2.46	5.85
	MSC+1st Der+SG	4	1.88	1.00	1.38	14.66	1.88	0.99	1.37	9.46
	MSC+1st Der	5	1.59	1.00	1.25	17.34	2.03	0.99	1.59	8.75
	MSC+2nd Der+SG	3	1.60	1.00	1.27	17.24	2.62	0.98	1.97	6.91
	MSC+2nd Der	3	1.33	1.00	1.03	20.69	2.57	0.98	2.03	6.97
	MSC	5	2.44	0.99	1.77	11.29	2.22	0.98	1.73	8.01
	SNV+1st Der+SG	3	2.32	0.99	1.74	11.87	2.17	0.98	1.55	8.22
	SNV+1st Der	4	1.84	1.00	1.34	15.00	1.88	0.99	1.33	9.44
	SNV+2nd Der+SG	2	2.45	0.99	1.86	11.23	2.56	0.98	1.96	6.92
	SNV+2nd Der	3	1.30	1.00	1.01	21.20	2.59	0.98	2.03	6.92
	SNV	4	2.64	0.99	2.04	10.44	2.48	0.98	2.06	7.20

## Data Availability

The original contributions presented in the study are included in the article/Supplementary Materials; further inquiries can be directed to the corresponding author.

## Supplementary Materials

The following supporting information can be downloaded at https://www.mdpi.com/article/10.3390/foods15203690/s1, Table S1: Information on experimental samples; Table S2: Experimental sample design; Table S3: Comparison table of predictive capabilities of different modeling methods; Table S4: Composition of the sample datasets used for the five material-specific PLSR models; Table S5: Overall and class-wise classification performance of the machine-learning models.

## Abbreviations

The following abbreviations are used in this manuscript:

ZSS	Ziziphi Spinosae Semen
ZMS	Ziziphi Mauritianae Semen
HD	*Hovenia dulcis* Seed
LCS	*Lens culinaris* Seeds
AB	Adzuki Bean
SRB	Scarlet Runner Bean
FT-NIR	Fourier Transform Near-Infrared Spectroscopy
PCA-X	Principal Component Analysis of the X Matrix
PLS-DA	Partial Least Squares Discriminant Analysis
OPLS-DA	Orthogonal Partial Least Squares Discriminant Analysis
OSC	Orthogonal Signal Correction
SVM	Support Vector Machine
DA	Discriminant Analysis
ANN	Artificial Neural Network
KNN	K-Nearest Neighbor
DD-SIMCA	Data-Driven Soft Independent Modeling of Class Analogy
PLSR	Partial Least Squares Regression
KS	Kennard–Stone Algorithm
MSC	Multiplicative Scatter Correction
SNV	Standard Normal Variate
SG	Savitzky–Golay Smoothing
1st Der	First Derivative
2nd Der	Second Derivative
LV	Latent Variables
RMSECV	Root Mean Square Error of Cross-Validation
RMSEC	Root Mean Square Error of Calibration
RMSEP	Root Mean Square Error of Prediction
MAE	Mean Absolute Error
RPD	Ratio of Performance to Deviation

## References

[1] Gong L., Xie J.-B., Luo Y., Qiu Z.-D., Liu J.-R., Mei N.-J., Chen Z.-Y., Wang F.-L., Huang Y., Guo J. (2023). Research Progress of Quality Control for the Seed of Ziziphus Jujuba Var. Spinosa (Bunge) Hu Ex H.F. Chow (Suan-Zao-Ren) and Its Proprietary Chinese Medicines. J. Ethnopharmacol..

[2] Zhang M., Liu J., Zhang Y., Xie J. (2022). Ziziphi Spinosae Semen: A Natural Herb Resource for Treating Neurological Disorders. Curr. Top. Med. Chem..

[3] Yan Y., Shen C., Pei X., Li A., Du H., Qin X., Du C. (2020). Compare the Chemical Profiling of Ziziphi Spinosae Semen and Ziziphi Mauritianae Semen Using Untargeted Metabolomics. J. Food Compos. Anal..

[4] Popstoyanova D., Gerasimova A., Gentscheva G., Nikolova S., Gavrilova A., Nikolova K. (2024). Ziziphus Jujuba: Applications in the Pharmacy and Food Industry. Plants.

[5] Xia F.-L., Cao H.-W., Gao L.-X., Kong Q., Li M., Wang C., Niu Y. (2023). Identification of Ziziphi Spinosae Semen from Its Adulterants Based on RAPD. Pharmacogn. Mag..

[6] Guo X., Li H., Feng H., Qi H., Zhang L., Xu W., Wu Y., Wang C., Liang X. (2021). Quality Analysis of Ziziphi Spinosae Semen Extracts Based on High Performance Liquid Chromatography Quantitative Fingerprint and Ultra-High Performance Liquid Chromatography-Tandem Mass Spectrometry Quantification. Se Pu.

[7] Sun S., Liu H., Xu S., Yan Y., Xie P. (2014). Quality Analysis of Commercial Samples of Ziziphi Spinosae Semen (Suanzaoren) by Means of Chromatographic Fingerprinting Assisted by Principal Component Analysis. J. Pharm. Anal..

[8] Beć K.B., Grabska J., Huck C.W. (2020). Near-Infrared Spectroscopy in Bio-Applications. Molecules.

[9] Zhang C., Su J. (2014). Application of near Infrared Spectroscopy to the Analysis and Fast Quality Assessment of Traditional Chinese Medicinal Products. Acta Pharm. Sin. B.

[10] Zhang Z.-Y., Wang Y.-J., Yan H., Chang X.-W., Zhou G.-S., Zhu L., Liu P., Guo S., Dong T.T.X., Duan J.-A. (2021). Rapid Geographical Origin Identification and Quality Assessment of Angelicae Sinensis Radix by FT-NIR Spectroscopy. J. Anal. Methods Chem..

[11] Chen H., Tan C., Li H. (2020). Untargeted Identification of Adulterated Sanqi Powder by Near-Infrared Spectroscopy and One-Class Model. J. Food Compos. Anal..

[12] Li S., Xing B., Lin D., Yi H., Shao Q. (2020). Rapid Detection of Saffron (*Crocus sativus* L.) Adulterated with Lotus Stamens and Corn Stigmas by near-Infrared Spectroscopy and Chemometrics. Ind. Crops Prod..

[13] Rodionova O.Y., Titova A.V., Pomerantsev A.L. (2016). Discriminant Analysis Is an Inappropriate Method of Authentication. TrAC Trends Anal. Chem..

[14] Strani L., Cocchi M., Tanzilli D., Biancolillo A., Marini F., Vitale R. (2025). One Class Classification (Class Modelling): State of the Art and Perspectives. TrAC Trends Anal. Chem..

[15] Kucheryavskiy S., Rodionova O., Pomerantsev A. (2024). A Comprehensive Tutorial on Data-driven SIMCA: Theory and Implementation in Web. J. Chemom..

[16] Fan Z.-L., Li Q., Zhang Z.-T., Bai L., Pu X., Shi T.-W., Chai Y.-H. (2026). Authenticity Identification and Quantitative Analysis of Dendrobium Officinale Based on Near-Infrared Spectroscopy Combined with Chemometrics. Foods.

[17] Li M.-X., Shi Y.-B., Zhang J.-B., Wan X., Fang J., Wu Y., Fu R., Li Y., Li L., Su L.-L. (2023). Rapid Evaluation of Ziziphi Spinosae Semen and Its Adulterants Based on the Combination of FT-NIR and Multivariate Algorithms. Food Chem. X.

[18] Wan X., Fu R., Zhu W.-H., Li M.-X., Guo Q., Li Y., Yu H.-R., Qiao J., Zheng Y.-Y., Lu T.-L. (2026). Research on the Identification of Ziziphi Spinosae Semen and Its Adulterants Based on the Fusion of Hyperspectral Technology and Transfer Learning. Spectrochim. Acta A Mol. Biomol. Spectrosc..

[19] Zheng C., Li J., Liu H., Wang Y. (2023). Data Fusion of FT-NIR and ATR-FTIR Spectra for Accurate Authentication of Geographical Indications for Gastrodia Elata Blume. Food Biosci..

[20] Bylesjö M., Rantalainen M., Cloarec O., Nicholson J.K., Holmes E., Trygg J. (2006). OPLS Discriminant Analysis: Combining the Strengths of PLS-DA and SIMCA Classification. J. Chemom..

[21] Balabin R.M., Safieva R.Z., Lomakina E.I. (2010). Gasoline Classification Using near Infrared (NIR) Spectroscopy Data: Comparison of Multivariate Techniques. Anal. Chim. Acta.

[22] Xu Y., Goodacre R. (2018). On Splitting Training and Validation Set: A Comparative Study of Cross-Validation, Bootstrap and Systematic Sampling for Estimating the Generalization Performance of Supervised Learning. J. Anal. Test..

[23] Westad F., Marini F. (2015). Validation of Chemometric Models—A Tutorial. Anal. Chim. Acta.

[24] Pagani A.P., Camargo G., Ibañez G.A., Olivieri A.C., Pomerantsev A.L., Rodionova O.Y. (2024). Data-Driven Version of Multiway Soft Independent Modeling of Class Analogy (N-Way DD-SIMCA): Theory and Application. Anal. Chem..

[25] Xu Y., Zomer S., Brereton R.G. (2006). Support Vector Machines: A Recent Method for Classification in Chemometrics. Crit. Rev. Anal. Chem..

[26] Marini F. (2010). Classification Methods in Chemometrics. Curr. Anal. Chem..

[27] Marini F. (2009). Artificial Neural Networks in Foodstuff Analyses: Trends and Perspectives a Review. Anal. Chim. Acta.

[28] Todeschini R. (1989). K-Nearest Neighbour Method: The Influence of Data Transformations and Metrics. Chemom. Intell. Lab. Syst..

[29] Chen P., Shao X., Wen G., Song Y., Fu R., Xiao X., Lu T., Zhou P., Guo Q., Shi H. (2024). Computer Vision-Based Fire–Ice Ion Algorithm for Rapid and Nondestructive Authentication of Ziziphi Spinosae Semen and Its Counterfeits. Foods.

[30] Sharma A., Sogarwal P., Kumar A., Ramesh (2025). Use of Near-Infrared Spectroscopy for Screening the Oil Content, Protein, Phytic Acid, Glucosinolates, and Fatty Acid Profile in Oilseed Brassica Species. Front. Nutr..

[31] Moll V., Beć K.B., Handle L., Huck C.W. (2025). Wavelength-Specific Information in Benchtop and Handheld Vis-NIR and UV–Vis Spectroscopy: The Case of Coffea Arabica. Food Res. Int..

[32] Chen Y., Ozaki Y., Czarnecki M.A. (2013). Molecular Structure and Hydrogen Bonding in Pure Liquid Ethylene Glycol and Ethylene Glycol–Water Mixtures Studied Using NIR Spectroscopy. Phys. Chem. Chem. Phys..

[33] De Girolamo A., Cervellieri S., Mancini E., Pascale M., Logrieco A.F., Lippolis V. (2020). Rapid Authentication of 100% Italian Durum Wheat Pasta by FT-NIR Spectroscopy Combined with Chemometric Tools. Foods.

[34] Beganović A., Moll V., Huck C.W. (2019). Comparison of Multivariate Regression Models Based on Water- and Carbohydrate-Related Spectral Regions in the near-Infrared for Aqueous Solutions of Glucose. Molecules.

[35] Geladi P., MacDougall D., Martens H. (1985). Linearization and Scatter-Correction for near-Infrared Reflectance Spectra of Meat. Appl. Spectrosc..

[36] Zhao J., Zhou Z., Zhao F., Yan X., Pan J., Qu H. (2022). Development and Validation of Global Prediction Models for Monitoring the Manufacturing Process of Herbal Medicine by Ultraviolet Spectroscopy. Acupunct. Herb. Med..

